# Should Research on the Nutritional Potential and Health Benefits of Fermented Cereals Focus More on the General Health Status of Populations in Developing Countries?

**DOI:** 10.3390/microorganisms5030040

**Published:** 2017-07-25

**Authors:** Caroline Laurent-Babot, Jean-Pierre Guyot

**Affiliations:** NUTRIPASS—IRD, University of Montpellier, Montpellier SupAgro—911, avenue Agropolis, F-34394 Montpellier CEDEX 5, France; caroline.laurent@univ-montp2.fr

**Keywords:** lactic acid bacteria, malnutrition, low-income countries, infection, inflammation, oxidative stress, antioxidant

## Abstract

Cereal foods fermented by lactic acid bacteria are staples in many countries around the world particularly in developing countries, but some aspects of the nutritional and health benefits of traditional fermented foods in developing countries have not been sufficiently investigated compared to fermented foods in high-income countries. Today, malnutrition worldwide is characterized by a double burden, excess leading to non-communicable diseases like obesity or diabetes alongside micronutrient deficiencies. In addition, populations in developing countries suffer from infectious and parasitic diseases that can jeopardize the health benefits provided by their traditional fermented foods. Using examples, we argue that research on traditional fermented cereals in developing countries should focus more on their effect on inflammation and oxidative stress under conditions including infectious or non-infectious gut inflammation.

## 1. Introduction

Cereals are the main staples in developing countries where sources of dairy products and meat are limited. Statistics comparing the consumption of the 223 most consumed food categories in Europe and Africa between 2001 and 2011 showed that, in Africa, cereal products are 1- to 9-fold more available (409 g/capita/day) than any other food categories, followed by starchy roots and tubers [1]. Cereals are consistently the main crop in Africa, and are cultivated on 45% of all arable land. For more information on the production and sale of cereals in Africa, see Galati et al. [2]. Unfortunately, statistics that are more focused on the production and consumption of cereal-based foods fermented by lactic acid bacteria (LAB) in developing countries are not available in authorized statistical databases. However, anyone who has travelled or worked in these countries most probably encountered, whether consciously or not, different kinds of cereal-based fermented foods among other types of fermented foods (meat, fish, etc.).

Many review and research articles have focused on different aspects of the nutritional and health benefits of lactic acid fermented foods lato sensu, often linked with the health concerns of the population in high income countries, including probiotic effects, allergy, and intolerance to food components, bioactive compounds due to the microbial action [3,4,5]. Browsing the Web of Science^TM^ shows that most of these papers deal with the health effects of dairy products but comparatively few with the benefits of vegetable-based fermented products and even fewer with LAB-fermented cereals. Many nutritional and health benefits are assumed in LAB-fermented cereal foods [6], e.g., increased protein and starch digestibility (whose effects may be good or bad depending on the age class), or increased vitamin content. The positive effects are too often generalized and cited without scientific evidence, with the exception of breads made from wheat, rye, and barley sourdough in Europe, whose health effects have been investigated in detail [7,8,9,10]. Due to the variety of cereals, i.e., maize, sorghum, pearl millet, rice, and wheat grown, and of processing methods used around the world [11], we think it is better not to make generic health claims about this category of foods, which requires very complex and challenging research.

The aim of this paper is not to review topics related to LAB-fermented cereal foods, which have already been extensively reviewed, particularly in the case of wheat, rye, and oat sourdough [7,8,12,13,14]. Investigations on these sourdoughs, mainly conducted by European research groups, have also identified the need for research on fermented cereal foods in developing countries. Rather, our aim here is to ask how research on LAB-fermented cereal foods in developing countries can tackle the more general problem expressed by international organizations concerned with global nutrition (WHO, FAO, IFPRI), by first describing the international priorities related to malnutrition and then by focusing on aspects that have been poorly explored to date, such as the influence of consuming LAB-fermented cereals on systemic and gut inflammation and on oxidative stress. At first glance, it could seem to be beyond the scope of a short position paper dealing with the health benefits of cereal fermentations to provide an overview of the world nutritional status; however, given the infectious status of populations in developing countries, we believe nutritional status should be the guiding principle for research into the health benefits of lactic acid fermentations lato sensu and cereal fermentation in particular.

## 2. Malnutrition Worldwide: Priorities

According to the 2016 Global Nutrition Report [15], out of a world population of 7 billion, about 2 billion suffer from micronutrient malnutrition and nearly 800 million from calorie deficiency. Iron deficiency is the most common cause of anemia, although other conditions, such as folate, vitamin B12, and vitamin A deficiencies, chronic inflammation, parasitic infections, and inherited disorders can all cause anemia in addition to other health disorders. For instance, nearly 250 million preschool children suffer from vitamin A deficiency. Among other benefits, vitamin A is essential for the normal functioning of the visual system [16]. Mineral deficiencies in cereal fermented foods and methods to improve their bioaccessibility are relatively well covered [17] and are not discussed per se in this paper, except in relation with the infection status of the populations of developing countries. Despite some general claims, there is a surprising lack of research on the effect of lactic acid fermentation on the vitamin content of LAB-fermented cereal foods. Although it is known that LAB can be auxotrophic or prototrophic, we do not know if LAB really are able to increase the vitamin content of cereals either by natural fermentation or through more sophisticated methods e.g., starter cultures. Recently, a review of folate production by LAB in fermented cereals [18] addressed this issue and a recent work showed that, despite the high genetic potential for folate synthesis in LAB in *ben-saalga* (a pearl-millet fermented porridge from Burkina Faso), the folate content of this food was very low [19]. In addition, other potential nutritional benefits discovered by gene screening of LAB and metagenomes from *ben-saalga*, samples still have to be linked with the real nutritional quality of the food [20].

Another striking fact is that, according to the 2016 Global Nutrition report [15], out of 5 billion adults, 2 billion are overweight or obese. Among the 667 million children under five worldwide, 159 million are stunted, 50 million are wasted and 41 million are overweight [15]. The nutritional transition is characterized by the increasing prevalence of obesity, insulin resistance, and cardiovascular diseases among populations. These non-communicable (chronic) diseases are not only found in high-income countries but unfortunately also in many developing countries including in North Africa and sub-Saharan Africa.

## 3. Infectious Status, Gut Inflammation in Populations of Developing Countries

The above statistics also need to be considered in relation with the intestinal infectious status of the populations of developing countries and vector-borne diseases. The following citation from the African Regional Health WHO Report [21] sums up the situation very well: “Infants who have survived the neonatal period are vulnerable to infectious diseases, especially lower respiratory tract infections, diarrheal disease, malaria, measles, and HIV. These children may also lack access to nutritious food, which increases their risk of developing diseases.”

Infant death related to diarrhea decreased worldwide between 2003 (1,008,470 infant deaths) and 2015 (525,977) according to WHO statistics (WHO “Global Health Observatory data repository”) [22]. Nevertheless, diarrheal diseases accounted for 6% of deaths in sub-Saharan Africa in 2012 [23]. Infections may be due to rotavirus, pathogenic bacteria (*Escherichia coli*, *Campylobacter jejuni* and *Shigella*), and helminthic parasites. However, a very interesting recent investigation into the environmental enteric dysfunction hypothesis showed that most stunting cannot be explained by poor diet or by diarrhea but rather by subclinical gut disease due to microbes ingested from unhygienic environments, which induce chronic immune activation [24].

Therefore, research on malnutrition should also take the infectious status of populations of developing countries into account. The chronic infectious status of poor and at risk populations in these countries could limit the potential benefits of eating fermented foods or impair strategies to improve their quality. If we take as an example, iron fortification of foods (whether fermented or not) or hydrolysis of chelating molecules to improve iron bioaccessibility, in some cases, the infectious status of the gastrointestinal tract (e.g., leading to diarrheas) means that efficient iron scavenging pathogenic microorganisms could outcompete absorption of iron through the cell intestinal layer and cause intestinal inflammation [25,26,27]. Increased iron availability also boosts the growth of *Plasmodium falciparum* malaria [28] and other parasitic diseases such as hookworm infection [29]. In such contexts (e.g., in countries where malaria is endemic), the first research question should thus be whether the impact of LAB-fermented foods through hydrolysis of chelating factors and improved iron bioaccessibility is negative or positive.

Most available data on non-infectious gut inflammation and barrier dysfunction concern the use of sourdough in bread making. Sourdough is used in wheat and rye fermentations for gluten detoxification by LAB proteolysis to reduce intestinal inflammation in celiac patients [7,10,30]. LAB can also act as probiotics in the gut and have a beneficial effect on intestinal barrier dysfunction. Some LAB, such as lactobacilli and bifidobacteria from West African fermented cereals, have been shown to have anti-inflammatory properties in an induced enterocolitis mice model [31,32,33,34,35]. It was shown that anti-inflammatory properties could be linked to the production of glutathione or superoxide dismutase (SOD) [36], as well as to the production of anti-inflammatory cytokines (IL 10, IFNγ) [37] or to the reduction of some mediators of inflammation such as TNFα or NO [36].

It is also important to recall that the link between inflammation and oxidative stress is well established. The production of oxygen free radicals (OFR) is implicated in the pathogenesis of a wide variety of diseases and plays a role in inflammation linked with the nutritional and infectious status of populations. Oxidative stress occurs during acute inflammatory states (infection, systemic inflammatory response syndrome) or chronic inflammatory syndromes (inflammatory bowel disease, environmental enteric dysfunction, metabolic syndrome) [38,39]. Thus, depending on the situation, oxidative stress can be both the cause or the consequence of inflammation [40] and preventing or controlling the production of OFR by antioxidants could be a way to improve the health and nutritional status of at risk populations. In this context, despite the current lack of information, we now focus on the potential capacity of LAB-fermented cereal products to counteract oxidative stress and inflammation.

## 4. Antioxidant Activities of LAB-Fermented Cereal Products

Numerous studies have focused on the antioxidant capacity of traditional LAB-fermented foods and their effects on health [41,42,43]. Oxidative damage plays a significant pathological role in human disease and eating food with antioxidant capacity may benefit health. Among LAB-fermented foods, the most widely studied are the fermented soy foods consumed in traditional Asian diets. Fermentation with LAB led to better antioxidant activities, such as radical scavenging effects, ascorbate oxidation inhibition, ferric-reducing activity, than unfermented products [43,44,45].

Although fewer data are available on fermented cereal products, the ability of LAB fermentation to improve antioxidant activity is of increasing interest and a variety of mechanisms could be involved. First, fermentation could improve the concentration of antioxidant compounds, such as phenolic compounds, bioactive peptides or exopolysaccharides by microbial enzymatic reactions, leading to both the structural breakdown of plant cell walls and enzymatic hydrolysis of matrix compounds. However, another possible explanation is that the LAB themselves are capable of antioxidant activity. This is discussed in more detail below.

### 4.1. LAB as Antioxidants

One possible explanation for the enhanced antioxidant activity of LAB-fermented cereal products is that they are a good source of probiotics, which themselves may have antioxidant properties. Research on the probiotic characteristics of cereal-fermented foods is scarce. One such study is by Lin et Yen [46], who investigated the in vitro antioxidant activity of 19 strains of LAB by evaluating metal ion chelating availability, scavenging of reactive oxygen species, and inhibition of oxidative enzymes. All strains demonstrated reactive oxygen species scavenging ability and most, in particular *Streptococcus thermophilus* 821 and *Bifidobacterium longum* 15708, showed excellent metal ion chelating ability. Another such study is by Kim et al. [42], who investigated the in vitro antioxidant activity of some strains (*L. acidophilus* LA5, *L. casei* 01, *L. acidophilus* LA100, *L. bulgaricus* LB207 and *L. rhamnosus* GG744) and showed lipid peroxidation inhibition activity, hydroxyl radical scavenging activity, and ferrous iron chelating activity that varied with the strain.

Several mechanisms could explain the antioxidant properties of LAB, as reported in a review by Spyropoulos et al. [47]. First, some LAB including *Lactobacillus gasseri*, *L. plantarum*, *L. fermentum*, *Lactococcus lactis*, and *Streptococcus thermophilus* may reinforce the inherent cellular antioxidant defense by secreting antioxidant enzymes like superoxide dismutase (SOD) [33,48,49,50]. These LAB can also release and promote the production of glutathione (GSH), the main non-enzymatic antioxidant and free-radical scavenger [34,35,36].

LAB also promote the production of certain antioxidant biomolecules, for example, exopolysaccharides (EPSs). Various health benefits have been attributed to EPSs. Reinforcing this hypothesis, one study reported significant in vitro antioxidant and free-radical scavenging activities of the non-LAB *Bacillus coagulans* RK-02 via EPS production, compared to standard antioxidants such as vitamin C and vitamin E [51]. Finally, LAB may also exhibit metal chelating activities [46]. All of these data suggest that the LAB present in fermented cereals foods could play a therapeutic role in ROS-characterized gastrointestinal disorders by acting as probiotics.

### 4.2. LAB-Fermented Cereals as a Source of Phenolic Compounds

As mentioned above, fermentation of cereals by different strains of LAB can increase concentrations of small phenolic compounds such as phenolic acids or flavonoids. During fermentation, microbial enzymes can disintegrate the plant cell wall matrix, leading to the release of bound phenolic compounds but also to the depolymerization of high molecular weight phenolics [52]. For example, fermentation of flours made from whole grain barley and oat groat using probiotic LAB strains (*Lactobacillus johnsonii* LA1, *Lactobacillus reuteri* SD2112, and *Lactobacillus acidophilus* LA-5) can significantly increase the concentration of free phenolic acids, thereby improving their bioavailability [53].

The enhancement of phenolic compounds in fermented cereals could be linked to an increase in their antioxidant capacity. In an example from a different type of raw material, spontaneous and induced fermentations by *Lactobacillus plantarum* was shown to improve the concentration of phenolic compounds in cowpea flour, leading to increased antioxidant activity, free radical scavenging activity [54]. p-coumaric, caffeic, ferulic and m-coumaric acids are also metabolized by *L. plantarum* [55].

Dordevic et al. [56] showed that fermentation of several cereals (buckwheat, barley, wheat, rye) by *Lactobacillus rhamnosus* for 24 h increased total phenolic content and antioxidant activities measured by diphenyl-2-picryl-hydrazyl (DPPH) radical scavenging activity, ferric ion-reducing antioxidant power (FRAP), and lipid peroxidation inhibition ability. Other authors previously demonstrated that barley and buckwheat contained substantial amounts of phenolic antioxidants that effectively scavenged free radicals, especially peroxyl, DPPH, and hydroxyl radicals [57,58]. In the above-mentioned study by Dordevic et al., fermentation of these cereals enhanced their antioxidant properties compared to unfermented samples. In rye, fermentation has been shown to increase antioxidant activity (DPPH radical scavenging activity) in the methanol extracted fraction of rye sourdough, concurrently with increasing levels of easily extractable phenolic compounds [59]. Chung et al. [60] showed in vitro antioxidant activities in ethanol extracted from fermented red beans by *Bacillus subtilis* IMR-NK1, including radical scavenging activities and Fe^2+^-chelating activity.

More recently, Wang et al. [61] studied the effect of fermentation of certain cereals including adlay, shelled chestnuts, lotus seeds, and shelled westnuts with *B. subtilis* or *L. plantarum* on antioxidant properties. Fermented cereals displayed better free radical-scavenging activities, improved ferric-reducing and chelating Fe^2+^ abilities, and increased concentrations of phenolics and flavonoids.

Although studies on traditional fermented cereals in developing countries are rare, one could deduce that the capacity of LAB fermentation to improve antioxidant activities via polyphenol content may be similar.

### 4.3. LAB-Fermented Cereals as a Source of Bioactive Peptides

Bioactive peptides in food are amino-acid chains encrypted in a protein sequence that, once the peptides are released, exhibit several bio-functionalities and may play diverse therapeutic roles in the human body. These peptides may already be present in foods as natural components or may result from hydrolysis by chemical or enzymatic treatments (digestion, hydrolysis, or fermentation).

Numerous biological activities of peptides derived from dairy proteins, including immunomodulatory, antioxidant, or antihypertensive activities are reviewed by Korhonen and Pihlanto (2003) [62], and the protective role of these peptides in intestinal barrier dysfunction such as inflammatory bowel disease or metabolic syndrome reviewed by Martínez-Augustin et al. (2014) [63].

Although most of the bioactive peptides studied to date are derived from milk, it is important to bear in mind that any source of protein, including cereals or legumes, can harbor bioactive peptides, in particular during fermentation, although studies of this pathway are rare. Cereals are known to be an important source of bioactive peptides and two major reviews recently focused on the capacity of some cereals (wheat, barley, corn, rice) and pseudocereals (buckwheat, amaranth) to release bioactive peptides with anticancer, anti-inflammatory, antioxidant, and cardiovascular protective properties [64,65].

The enzymatic activity of LAB largely contribute to the release of bioactive peptides, either into the food matrix (starter or autochthonous LAB) or in the gut (endogenous microbiota or probiotics) in particular from fermented dairy products.

Some studies revealed promising effects of bioactive peptides derived from fermented cereals. For example, fermentation of cereal and pseudo-cereal flours (wholemeal wheat, soybean, barley, amaranth, and rye flour) with sourdough LAB selected for their proteolytic activity successfully increased the concentration of the anti-cancer peptide lunasin [66]. Lunasin is probably the most widely studied peptide for its anticancer activities [67].

γ-Aminobutyric acid (GABA) is attracting increasing interest as a neurotransmitter and has been shown to have anti-hypertensive, diuretic, tranquilizer, and diabetes preventive effects [68]. Bacteria can produce GABA through decarboxylation of glutamate through glutamate decarboxylase (GAD). Lactobacilli are the best producers of GABA, but lactococci*, s*treptococci, and bifidobacteria can also synthesize GABA [69]. Coda et al. [30] used *L. plantarum* and *Lactococcus lactis* for sourdough fermentation of wheat, rye, spelt, oat, buckwheat, rice, amaranth, millet, chickpea, soy, and quinoa flours to make a functional bread enriched in GABA.

The production of GABA has also been demonstrated in rice bran extracts fermented with the LAB *Lactobacillus sakei* B2-16 [70]. Rizzello et al. [71] highlighted increased synthesis of GABA in sourdough fermentation of cereals by LAB, in particular in wholemeal wheat sourdough.

Lactic fermentation of buckwheat sprouts was shown to produce new peptides with significant blood pressure-lowering effects in hypertensive rats and to increase active GABA and tyrosine compounds already present in buckwheat sprouts [72].

On the other hand, it has been shown that a pool of selected LAB had the capacity to release antioxidant peptides from some cereal flours like spelt and kamut in appropriate sourdough fermentation conditions [73]. The ex vivo antioxidant activity of these peptides, tested on mouse fibroblasts artificially subjected to oxidative stress, was comparable to that of α-tocopherol [73].

Fermentation of cereals by LAB could thus improve their natural bioactivity by increasing antioxidant activities through several different processes, thereby providing more benefits to human health. However, it should be noted that many of these studies were conducted using ethanol extracted from fermented cereal products and not the whole cereal product itself. What is more, as Hur et al. [41] recently pointed out in their review focused on fermented plant-based foods, various factors can influence the increase in antioxidant activity, including the microorganism, pH, temperature, fermentation time, the kind of food, and aerobic conditions, thereby underlining the need for further investigation.

## 5. Conclusions

To conclude this opinion paper, we would like to emphasize the influence that consuming LAB-fermented cereals could have on oxidative stress and inflammation either by modifying the food matrix or through the potential action of probiotic LAB. On this last point, it should be noted that many of these foods are cooked before eating, probably limiting their impact on the health of the host, since, by definition, probiotics must be living organisms.

We also emphasize the need to investigate the interplay between the food matrix, food and gut microbiota. It is clear that such sophisticated investigations will require more collaborative research programs between high- and low-income countries.
